# Accelerated Intermittent Theta Burst Stimulation Combined with Balance Training Improves Balance in an Individual with Corticobasal Syndrome

**DOI:** 10.1002/mdc3.70213

**Published:** 2025-07-02

**Authors:** Karishma R. Ramdeo, Stevie D. Foglia, Malaikah Ahmad, Nafia Al‐Mutawaly, Justin Lee, Robert Chen, Aimee J. Nelson

**Affiliations:** ^1^ Department of Kinesiology McMaster University Hamilton Ontario Canada; ^2^ School of Biomedical Engineering McMaster University Hamilton Ontario Canada; ^3^ School of Electrical Engineering Technology McMaster University Hamilton Ontario Canada; ^4^ Faculty of Medicine McMaster University Hamilton Ontario Canada; ^5^ Krembil Research Institute University Health Network Toronto Ontario Canada

**Keywords:** accelerated intermittent theta burst stimulation, balance, cognition, corticobasal syndrome, rTMS

Intermittent theta burst stimulation (iTBS) is an effective intervention for balance recovery when delivered over primary motor cortex (M1).^1^ Accelerated iTBS (aiTBS) delivers multiple sessions of iTBS spaced approximately one‐hour apart.^2^ aiTBS has antidepressant efficacy in major depressive disorder^3^ and improves memory in Alzheimer's disease.^3^ Here, we combine aiTBS with balance training. aiTBS was intended to prime M1 to enhance the opportunity for motor learning.^4^

Herein we report a 77‐year‐old female with corticobasal syndrome for 2 years, with left hand “alien limb” phenomenon, left‐hand dystonia, and sensory loss in left upper limb. At the time of diagnosis, the MoCA indicated 18/30. The individual was taking donepezil (10 mg), atorvastatin, telmisartan, furosemide, bisoprolol fumarate, empagliflozin, and acetylsalicylic acid.

Balance and cognition were measured 2 days before and following aiTBS and balance training (Fig. 1A). Using the BTracks System (Balance Tracking Systems Inc., USA), we assessed static balance impairments and fall risk. These data include the center of pressure (COP) path length from three 20‐second trials. The Limits of Stability protocol measures dynamic balance based on the size of the functional base of support. Cognitive performance was assessed using the Mini Mental State Examination (MMSE), Montreal Cognitive Assessment (MoCA) and Cornell Scale for Depression in Dementia (CSDD).^3^

**FIG. 1:**
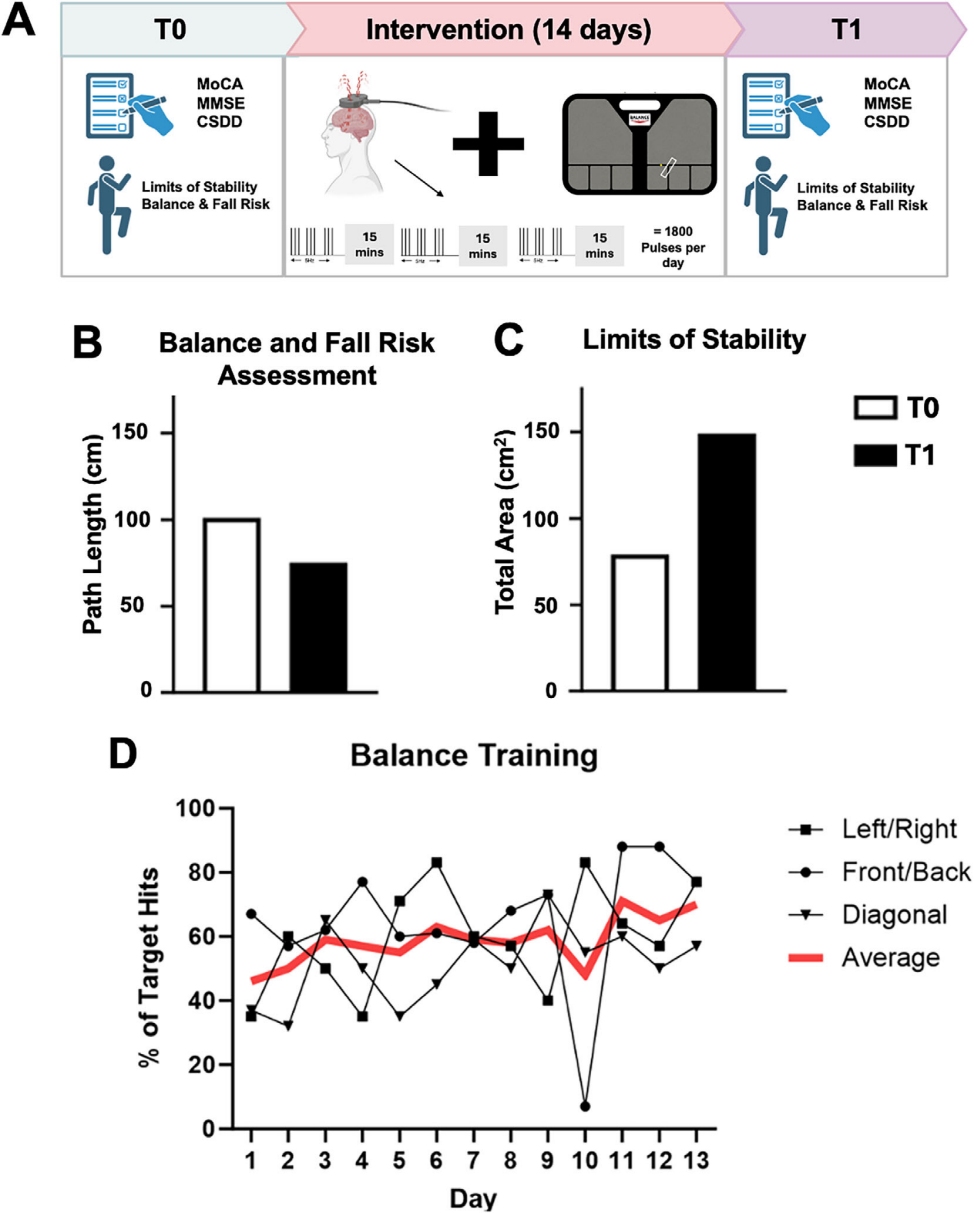
(A) aiTBS followed by 10 mins of balance training was performed for 14 consecutive days. Assessments of balance were obtained before (T0) and after (T1) training. (B) Balance and Fall Risk Assessment before (T0) and after intervention (T1) (Path length in cm). (C) Limits of stability before and following intervention (Total Area in cm^2^). (D) Average performance on balance training across conditions (left/right, front/back and diagonal direction) for each day of the intervention.

aiTBS was delivered over the motor hotspot of abductor pollicis brevis muscle representation of the left primary motor cortex, ipsilateral to upper limb exhibiting alien limb phenomenon and dystonia, (Magstim Rapid 2, UK) with 90 mm diameter figure‐eight coil. aiTBS delivered three iTBS sessions separated by 15‐minutes^3^ (Fig. 1A). iTBS consisted of 600 stimuli delivered in 50 Hz bursts of 3 pulses at 80% of the resting motor threshold, (1800 stimuli per day)^3^ repeated for 14 consecutive days. Immediately following aiTBS, ~10 minutes of balance training was performed on the BTtracks system by having the individual move their COP to target zones displayed on the computer. Performance is based on the percentage of targets obtained within a session.

The participant completed all brain stimulation sessions (three sessions per day x 14 days) and 93% of balance training sessions. aiTBS was well tolerated. Results show an increase in the Limits of Stability by 88% (Fig. 1C) and a decrease in the Balance and Fall risk by 26% (Fig. 1B). A 35% increase in MMSE was observed following the intervention which exceeds the clinically meaningful improvement^5^ of 3 points (MMSE pre = 20, post = 27). Changes in MoCA are not clinically significant^5^ (MoCA pre = 18, post = 16). These data suggest improvements in attention‐related functions as reflected in MMSE without meaningful gains in executive or complex cognitive tasks as captured by MoCA. CSSD scores decreased by ~75% following the intervention (pre = 4, post = 1).

This is the first reported use of aiTBS in an individual with a Parkinson‐plus disorder. Limitations of the current research include the participant's use of donepezil, a cholinesterase inhibitor, which may influence cognitive scores. Additionally, the absence of a control condition limits the ability to isolate the effects of the intervention. Further, it would be beneficial to determine the longevity of the intervention by including a longer term follow up. This report provides insight into the potential clinical utility of aiTBS plus balance training to improve Parkinson‐plus disorder symptoms.

## Author Roles

Research project: A. Conception, B. Organization, C. Execution; Statistical Analysis: A. Design, B. Execution, C. Review and Critique; Manuscript Preparation: A. Writing of the first draft, B. Review and Critique;

K.R.: 1A, 1B, 1C, 2A, 2B, 2C, 3A, 3B.

S.F.: 3B.

M.A.: 1C, 3B.

N.A.‐M.: 3B.

J.L.: 3B.

R.C.: 3B.

A.N.: 1A, 1B, 1C, 2A, 2B, 2C, 3A, 3B.

## Disclosures

**Ethical Compliance Statement:** This research study was revied by the Hamilton Integrated Research Ethics Board. Written and oral consent was obtained for this work. We confirm that we have read the Journal's position on issues involved in ethical publication and affirm that this work is consistent with those guidelines.

**Funding Sources and Conflict of Interest:** This work was supported by Canada Research Chair funds to AJN. NA‐M is the president of Ressam Gardens Memory Care Community where the research was conducted.

**Financial Disclosure for the previous 12 months:** The authors declare that there are no additional disclosures to report.

## Conflict of Interest

NA‐M is the president of Ressam Gardens Memory Care Community where the research was conducted.

## Funding

This work was supported by Canada Research Chair funds to AJN.

## Data Availability

The data that support the findings of this study are available from the corresponding author upon reasonable request.
